# The Total Mercury Concentration in Organs of Eurasian Magpies (*Pica pica*) and Common Woodpigeons (*Columba palumbus*) from the Warsaw Municipal Area

**DOI:** 10.3390/ani13040575

**Published:** 2023-02-06

**Authors:** Ewa M. Skibniewska, Michał Skibniewski

**Affiliations:** 1 Department of Biology of Animal Environment, Institute of Animal Science, Warsaw University of Life Sciences, Ciszewskiego Street 8, 02-786 Warsaw, Poland; 2 Department of Morphological Sciences, Institute of Veterinary Medicine, Warsaw University of Life Sciences, Nowoursynowska Street 159, 02-776 Warsaw, Poland

**Keywords:** mercury, birds, Eurasian magpie, common woodpigeon, liver, kidney, muscles

## Abstract

**Simple Summary:**

The presence of pollutants, including heavy metals, in the environment is a threat to the health and life of living organisms. Mercury is an element widely distributed in the natural environment. It is released from natural sources, and due to anthropogenic activity as well. The present study analyzed the mercury content of liver, kidney, heart and muscle tissue in two species of birds from the Warsaw area, which were used to assess local environmental contamination with this metal. The results indicate that the tissue’s mercury content was at a low level that did not pose a health risk to the animals, and this finding is strictly associated with low environmental exposure. Considering the mercury content of the organs, they can be arranged in the following descending order: kidney > liver > heart > muscles.

**Abstract:**

Mercury is a toxic element widely distributed in the natural environment, affecting animals’ health. It is released into the environment from both natural and anthropogenic sources. The present study analyzed the mercury concentrations in liver, kidney, heart and muscle tissue in two species of birds from the Warsaw area, which were used as bioindicators of local environmental pollution with this metal. The mercury content in the examined samples was determined using atomic absorption spectrometry (AAS) utilizing automatic mercury analyzer type AMA 254. The highest mercury content was found in the body of Eurasian magpies, in which it was 0.025; 0.021; 0.006; 0.0037 and 0.029 mg kg^−1^ of tissue wet weight for kidney, liver, heart, thigh muscles and pectoral muscles, respectively. In the case of common woodpigeons, the content of this metal was significantly lower, amounting to 0.007; 0.005; 0.002; 0.001 and 0.001 mg∙kg^−1^ wet weight for kidney, liver, heart, thigh muscles and pectoral muscles, respectively. In light of data from the available literature, the values obtained should be considered low, not causing a risk to animal health. The results obtained indicate low environmental exposure to this element.

## 1. Introduction

Mercury (Hg) and its organometallic compounds are among the most toxic substances in terrestrial and aquatic ecosystems. In the environment, it originates both from natural circulation in the biosphere and from anthropogenic activities [1,2]. The circulation of mercury includes, among other things, its release from rocks and minerals, as well as atmospheric transport. Certain amounts of mercury are also evaporated from the surface of lands, seas and oceans [3,4,5].

Natural sources of mercury emissions to the environment are volcanic eruptions, forest fires, and the weathering and erosion of minerals such as cinnabar HgS or calomel Hg_2_Cl_2_, in the composition of which it is present [6,7]. The main sources of anthropogenic emissions include the combustion of fossil fuels (mainly coal and lignite), oil processing, gold and non-ferrous metal mining and processing, the metallurgical industry (including non-ferrous metallurgy), waste incineration, cement production, chlorine production, the use of mercury lamps, and industrial processes that use mercury and its compounds [8,9,10]. Mercury is also a constant component of municipal wastewater. In the 1970s, mercury seed dressings were an important source of this element in agricultural ecosystems. It is assumed that between 4400 and 8200 t of Hg_2_ are emitted to the environment annually. Recently this trend has not changed, although concentrations are tending to decrease slightly [7,11,12]. 

The main source of mercury emissions in Poland is the energy sector that uses coal combustion. It has been estimated that it is responsible for about 71% of emissions, whereas other industries account for 26% of national mercury emissions. In addition, there is the local impact of so-called low emissions caused by the use of coal as the main energy source for heating homes [7,9,13,14]. Its air concentrations in agricultural regions are most often at the levels of 2–6 μg m^−3^, whereas in large agglomerations its content ranges from 5 to 50 μg m^−3^. Mercury emitted into the atmosphere undergoes a number of transformations; these depend, among other things, on temperature, the degree of insolation, the chemical reactions in which this metal participates and the presence of other pollutants [9,10,13]. Like other heavy metals, mercury undergoes partial gradual incorporation into the biological cycle when released into the environment, posing a risk to humans and animals [15,16,17,18]. The toxicity and transport of mercury in the environment depends on its physicochemical form. In the environment, it occurs in three forms: elemental mercury, inorganic compounds (mainly mercuric chloride) and organic mercury (II) salts. The most harmful are the organometallic forms, methylmercury and phenyl-, ethyl- and methoxyethylmercury compounds [19,20,21,22]. Mercury does not fulfill any physiological functions. Its toxicity, combined with the ability to accumulate in tissues, generates a large health risk for many organisms, in particular because the element persists in the environment for a long time and undergoes biomagnification [6,23,24,25]. As a typical heavy metal, it binds selectively to the sulfhydryl groups of proteins making up cellular structures. Virtually all proteins contain sulfhydryl groups that react with mercury, so poisoning with this element can disrupt all enzymatic reactions and damage the proteins that make up cellular structures [20,26,27]. Mercury compounds impair almost all enzyme reactions, including protein biosynthesis processes, causing pathological changes in the nervous system (particularly in the brain), and cause a range of metabolic disorders, such as nephrological, immunological, cardiac, motor, fertility and genetic disorders. In extreme cases they can lead to death [28,29,30,31]. 

The ease of mercury distribution can result in its increased accumulation in the environment and, subsequently, in the bodies of animals and humans [27,32,33]. Mammals and birds are exposed to mercury, which even at subclinical levels affects their health and reproduction [32,34,35]. Among birds, predatory species, which are the last link in the trophic chain, are particularly vulnerable to mercury [36]. The risk of higher bioaccumulation of mercury in birds depends on the environment in which they live and their food base, as mercury enters their bodies mainly via the alimentary route [37,38,39,40]. Free-living birds are one of the important biomarkers of environmental pollution by this heavy metal. Mercury poisoning can cause a variety of pathological effects in birds, including behavioural changes, sluggishness, loss of appetite, problems with adequate moult, and imbalance limiting movement, which is particularly relevant for some migratory bird species [31,41,42,43]. Mercury’s ability to combine with a variety of constituents affects global environmental pollution even in areas remote from its emission sources [11,12,44].

The negative effects of mercury on human health have led to an increased interest in environmental quality, which has led to many studies on monitoring and assessing its pollution using, among others, birds [18,28,45]. Birds can accumulate large amounts of xenobiotics due to their position in the food chain and sensitivity to environmental changes, and they are often used as biomonitors of environmental pollution [29,38,41].

One way to assess mercury accumulation in wildlife species is to monitor long-term tissue residues, which reflect bioavailable fractions of mercury in the environment and can be used as a key indicator of adverse effects risk [6,46,47]. Despite many studies on the body mercury content of different bird species, relatively few have addressed the levels of this metal in internal tissues.

The aim of this study was to assess mercury accumulation in internal organs of free-living birds (Eurasian magpies and common woodpigeons) from the Warsaw area and to identify possible species differences in distribution of this metal in their bodies, which indirectly allows for assessment of the local environmental burden of this metal.

## 2. Materials and Methods

Research material was collected between 2016 and 2018 from two bird species: the Eurasian magpie (*Pica pica*), also known as the common magpie, and the common woodpigeon (*Columba palumbus*). The Eurasian magpie, belonging to the *Corvidae* family, is an omnivorous bird whose diet includes both plants and animal food, including dead animals left behind by predators and waste discarded by humans. The second species studied was the common woodpigeon (*Columba palumbus*), a migratory bird from the pigeon family (*Columbidae*). Due to changes brought about by human activity, the behavior of individuals of this species has also changed and now, in addition to migratory individuals, sedentary populations are increasingly found, since urban and suburban environments provide permanent food supplies and new opportunities for those birds. The common woodpigeon is the largest of the pigeon species found in Poland. Its diet varies seasonally; in early spring, birds mostly feed on shoots and seeds of wild plants, small fruits and snails. Towards the end of summer, their diet is dominated by cereal grains left in the fields.

The study material consisted of tissues taken from 40 dead birds (22 Eurasian magpies and 18 common woodpigeons), which were brought to veterinary clinics located in Warsaw. All birds were mature individuals. Some individuals died as a result of accidents, i.e., collisions with windows of buildings, or were hunted by pet cats and then picked up by pet owners. Dead specimens were left at veterinary clinics for disposal. Nevertheless, in most cases the cause of death remained unknown. Both species are monomorphic, so the sex of the animals was determined based on visual inspection of the gonads during dissection prior to material collection; hence only mature specimens were examined. Of the 18 common woodpigeons tested, 10 individuals were female, whereas of the 22 Eurasian magpies tested, female sex was recognized in 15 individuals. Only tissues from specimens with relatively well-preserved carcasses, without signs of decomposition or extensive damage to the common integument (allowing contamination of internal organs), were collected for determination of mercury concentrations. Soiling of the plumage in many individuals resulted in its exclusion from analytical procedures. Similarly, it was decided to not analyze the mercury content of the brain because several individuals had been decapitated, probably by animals, and several had signs of craniocerebral trauma, including bleeding into the eyeballs, ears, beak and nares and also cranial fractures or bruising, which in birds occurs after very severe head trauma resulting from collisions with, for example, glass panes or other hard obstacles. In view of this, it was decided not to collect brain tissue for testing. 

Based on the information obtained from the Third Local Ethics Committee in Warsaw, in light of the applicable legal regulations, tests using animal tissues collected post mortem do not require the obtainment of appropriate approval. Until analyses the material was stored in polyethylene containers in a deep-freeze state at a temperature of −20 °C.

The mercury content in the examined samples was determined using atomic absorption spectrometry (AAS) utilizing automatic mercury analyzer type AMA 254 by ALTEC Czech Republic, specifically designed to determine total mercury content without sample pretreatment or sample preconcentration. Additional homogenization and the special storage conditions of the samples are not necessary as well. AMA 254 uses an element-specific lamp that emits light at a wavelength of 253.7 nm, and a silicon UV diode detector for mercury quantitation. Samples were weighed (50 to 100 mg) with 0.1 mg readability with the use of an electronic analytical balance (model Radwag E425). Then, the analytical boat containing the sample was placed into the instrument in which automatic combustion was performed. The precision and accuracy of the method were previously evaluated using certified reference material (SRM; 1577c, bovine liver, National Institute of Standards and Technology NIST), and blanks were also run in each sample set. A recovery percentage of 91% ± 0.41 was obtained. About every 40 samples, a new boat was used after heating it twice to remove potential traces of mercury. The apparatus was calibrated using a polarographic mercury standard in 2% HNO_3_ (Merck, Darmstadt, Germany). The detection limits (LODs) obtained were 0.001 mg Hg kg^−1^. The concentration of mercury in the samples was presented in milligrams per 1 kg body weight (mg kg^−1^). Each measurement was replicated three times, and the result was expressed as the arithmetic mean of three measurements.

The statistical analysis of the results obtained, including the relationship in the individual groups, was developed using Statistica 13.3 software (TIBCO Inc.™ Palo Alto, CA, USA). The data distribution was analyzed using the Shapiro—Wilk *W* test followed by application of a non-parametric data analysis. In order to compare the differences between groups, the Mann—Whitney U test was used at the significance level of *p* ≤ 0.05 and *p* ≤ 0.01. The differences between the groups were analyzed using Spearman’s correlation coefficient at the significance level of *p* ≤ 0.05 and *p* ≤ 0.01.

## 3. Results

The organs of the common woodpigeons studied were characterized by lower average mercury concentrations than those of the Eurasian magpies. Nevertheless, it should be noted that in both species there were individuals whose mercury content was below the limit of detection (LOD) of the method used. Particularly among the pigeons, there were individuals in which even in the liver and kidney tissue no mercury was recorded. In the case of magpies, only skeletal muscles were characterized by relatively low mercury concentrations, since in the heart muscle the lowest value recorded was 0.001 mg kg^−1^ of wet weight. The basic statistical parameters for the mercury content of both species examined are presented in Table 1 and Table 2. 

In both species, the organ with the highest mercury content was the kidney, whereas the lowest average values were noted in pectoral muscles. The median values for the two types of skeletal muscle were similar, whereas the mercury content of the heart was significantly higher in both species studied. Small differences in mercury concentrations were observed between the kidney and liver, whereas differences between the kidney and pectoral muscles were at least several-fold. In the common woodpigeon it was 6.8 times, whereas in the Eurasian magpie it was as much as more than 8.6 times. 

**Figure 1:**
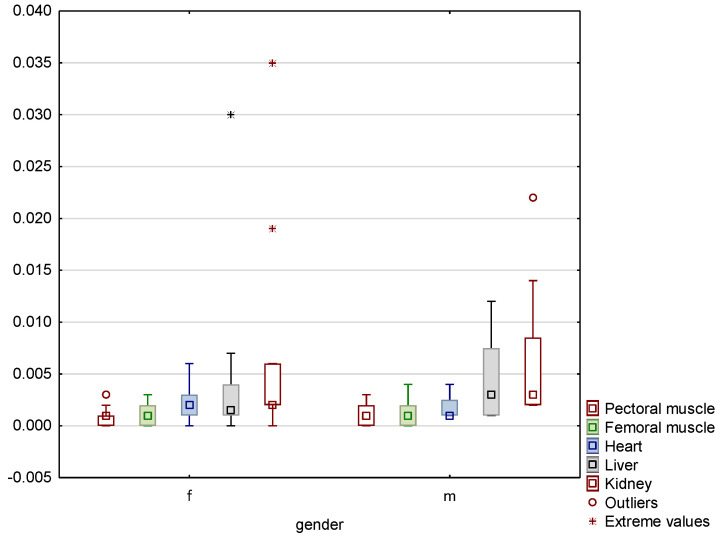
Box-and-whisker plot showing the median values for the mercury concentrations of the organs tested in common woodpigeons of both genders. Box shows 25–75% coefficient, whiskers show minimum and maximum range of non-outliers.

**Figure 2:**
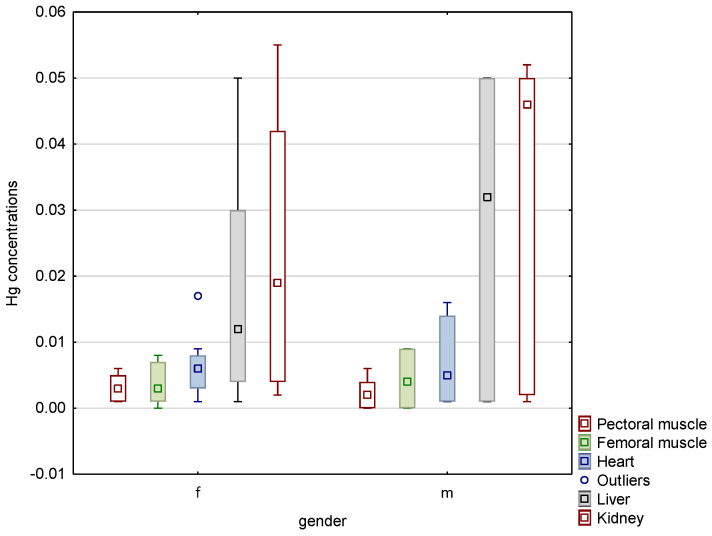
Box-and-whisker plot showing the median values for the mercury concentrations of the organs tested in Eurasian magpies of both genders. Box shows 25–75% coefficient, whiskers show minimum and maximum range of non-outliers.

Significant interspecies variations were noted, as confirmed by statistical testing. Median values for relevant organs in both species were significantly different at *p* ≤ 0.01. In an analysis of the effect of gender on the mercury content of bird tissues, it was found that in common woodpigeons, the median values for individual organs of individuals of both sexes did not differ significantly, as Figure 1 illustrates. Nevertheless, extreme values that were significantly above the values recorded in other individuals were recorded in the kidneys of two among ten females tested and the liver of one female. An outlier value was recorded in the kidney of one among eight male birds. With regard to the Eurasian magpies studied, it was found that the median values for the muscle tissue of individuals of both sexes did not differ significantly, whereas the mercury content of the liver and kidneys of males was higher than that of females. Data on the mercury content of the organs of this species are shown in Figure 2.

In both bird species studied, there were also highly significant correlations between the mercury concentrations of various organs, which confirms that the determination of mercury concentrations in the liver and kidney reflects the burden of this element on the whole body (Table 3 and Table 4).

## 4. Discussion

Using birds as bioindicators of mercury levels provides an important tool for studying environmental quality in various ecosystems [48,49]. Birds play an important role in food chains, have a wide geographic range, and are often sedentary species, so they are good local biomonitors for the presence of heavy metals [36,50,51]. Although the effects of mercury on various bird species have been studied extensively, the majority of them have been piscivorous, waterfowl or domesticated species [47,52]. Since recent studies revealed that mercury pollution not only plays a significant role in aquatic environments, but also affects terrestrial species, researchers’ attention has turned to other birds, including songbirds [49,53,54]. Given the expected worsening of mercury pollution, which affects more species than was previously thought, the authors of the present article are of the opinion that there are numerous synanthropic birds that can be successfully used in the study of urban as well as suburban environments. They include the common woodpigeon and common Eurasian magpie. Nevertheless, to date, data on their organ mercury concentrations is scarce and usually refers to the content of this metal in the feathers of the two species. Since it has been shown that feathers incorporate mercury in a dose-dependent way, many studies have been devoted to the analysis of this material and they have become an important biomarker for mercury concentration in birds. It is believed that mercury concentrations in feathers reflect approximately 70–93% of the total body burden of this element [55,56]. Although the relationship between mercury concentrations in feathers and body burden by this toxic element has been confirmed by numerous studies, the analysis of the mercury content of this biological matrix may be subject to some inaccuracy. This is mainly due to the morphological features and physiology of the feathers themselves, since their mercury concentration varies greatly even in the same individual, mostly due to different types of feathers. The second factor that should be taken into account in monitoring programs is moult, which affects mercury concentrations [6]. In addition, feathers are often contaminated by external deposits of numerous substances, including metals. Although many laboratory cleaning procedures have been proposed, none of them ensure complete purification of their surfaces from external metallic contaminants [57,58]. Therefore, authors of the present work decided not to include feather analysis in this study, since the material analyzed was obtained from dead individuals found at various places and stored in different conditions. There are no studies in the available literature on the tissue mercury content in the common woodpigeon and Eurasian magpie from the Warsaw municipal area. It is therefore not possible to directly relate the values obtained in our own research to the results of other researchers. The results obtained in our study show significant differences in mercury content both between the two species studied and between the average values for individual organs. Considering the mercury content of the organs, they can be arranged in the following descending order: kidney > liver > heart > muscles. This relationship applies to both species studied, but in magpies there is a difference in average values for limb muscles and pectoral muscles, whereas in pigeons the two muscle groups do not differ significantly in terms of the content of this element. It should be noted, however, that 3 individuals were present among the pigeons studied who had elevated mercury levels in liver and kidneys, reaching the highest value of 0.030 mg kg^−1^ and 0.035 mg kg^−1^ of organ fresh weight, respectively. The distribution of mercury concentrations among bird organs in our study is somewhat different than indicated in the literature, where liver content generally exceeds the renal concentration of this metal [59,60]. Results observed in our study may be due to the anatomical features of the birds in which the renal portal system is present. Venous blood draining from the pelvic limbs enters the renal portal veins, directing it to the capillary bed in these organs. It can be assumed that a certain mercury load enters the pelvic limb muscles, where the blood is subject to elimination by the renal portal system before it reaches the systemic circulation. However, considering the average values for both organs, it can be seen that the difference between the renal concentration of mercury and its content in pectoral muscles in the Eurasian magpie and common woodpigeon is 8.6 and 6.8 times higher, respectively. The difference between the kidneys and liver is not so significant, as both organs play a key role in methylmercury demethylation, thus playing a protective role against its toxic effects. In general, omnivorous magpies are characterized by higher mercury concentrations in all tissues studied compared to pigeons. Our results are in line with those reported by other authors, who stated that mercury accumulates in subsequent links of the food chain. Its level in birds depends primarily on the feeding base and increases in the following order: herbivores < omnivores < piscivores [61,62,63]. Although both bird species examined in this study inhabited the same areas, there are significant differences between them in their food bases. Common woodpigeons are classified as herbivores. In contrast, omnivorous Eurasian magpies are able to exploit different food sources found in cities, including anthropogenic food as well [64,65]. Moreover, the common woodpigeon population is limited by predators such as the Eurasian magpie, which is the main factor affecting the nesting success of the common woodpigeon in urban habitats. It was estimated that different corvids, including the Eurasian magpie, can significantly reduce common woodpigeon offspring in cities, especially during early spring, when they can eliminate more than 96% of them [66,67,68]. In light of the available data, it can therefore be said that, in some ways, the species studied are the next links in the trophic chain. Considering the data published to date, it can be concluded that the mercury content of bird tissues is within wide limits. In omnivorous terrestrial birds its median concentrations in parenchymatous internal organs ranged from 0.024 to 0.067 mg kg^−1^ wet weight [6,69]. Particularly low levels of mercury were found in muscle tissue, where it was repeatedly below the detection threshold [69]. Taking into account this background, the results obtained in our own research should be considered low, as they are close to the lower values recorded by the research quoted. The available literature practically lacks data on the species studied in term of their mercury concentrations and there are no reference values for mercury content in the internal organs of birds. Some authors have proposed a range within which they should fall. Puls [70] suggested that normal total mercury concentrations in the liver, kidneys and muscles of poultry were 0.01–0.1, 0.05–0.3 and 0.008–0.1 mg kg^−1^ wet weight, respectively. The values recorded in our study are far from total Hg concentrations associated with bird health impairments. Finley and Stendell [71] observed that in *Anas rubripes,* liver and kidney mercury concentrations at the levels of 23.0 and 16.0 µg g^−1,^ respectively, are associated with reproduction impairment in terms of decreased offspring survival. However, the values presented are very high. Shore et al. [72] proposed liver mercury concentration indicative of reproduction impairment at a significantly lower level, 2.0 mg kg^−1^ wet weight, and Zillioux et al. [73] suggested that a total mercury liver concentration of 5 mg kg^−1^ is associated with health and physiological processes impairment in water birds. Although liver, kidney and muscle tissue are believed to have a moderate priority for assessing bird mercury contamination since they cannot be obtained in non-invasive way, their advantage is the high correlation of results obtained with other tissues, including whole blood [74,75]. The total mercury concentrations in the liver and kidneys does not reflect methylmercury load, since the liver is strongly involved in the demethylation process and a large amount of inorganic mercury is removed via the kidneys [75]. For this reason, it is suggested that liver and kidney total mercury determinations should be accompanied by a chemical determination of methylmercury [76]. A certain solution may be the simultaneous determination of mercury in skeletal muscles, where its methyl derivatives are mainly deposited. Our results confirm the strong correlation of mercury content in the liver and kidney with other tissues. It is known that sensitivity to mercury differs widely among species [77]. We are not aware of data on the sensitivity of the species tested to mercury, including its methyl derivatives, and thus it is difficult to set threshold values above which the concentrations of mercury may negatively impact birds’ physiology. We are of the opinion that extending the study to more species inhabiting different areas will allow us to create a database of reference values for particular groups of birds.

## 5. Conclusions

Based on the results obtained, it can be concluded that tissue mercury concentrations in both bird species studied indicate low environmental exposure, and the values recorded should not pose a health risk for them. Although both species differ in their food base, some regularities in organ mercury concentrations have been observed; the highest values were recorded in kidney samples and the lowest in pectoral muscles. Distribution of mercury among organs in relation to birds’ feeding behavior indicates that the alimentary route plays the most important role in tissue contamination with this element. Despite the emerging summary studies on mercury content in various bird species, there is not sufficient information to create normal and toxicity benchmarks for them. Therefore, it seems that it is necessary to constantly add to the stock of available data relating to individuals representing species living in different habitats, which could be useful in environmental monitoring.

## Figures and Tables

**Table 1:** Mercury concentrations in organs of common woodpigeons in milligrams per kilogram of wet weight.

Organ Examined	N	Arithmetic Mean	Median	Min	Max	Q_1_	Q_3_	SD
Pectoral muscle	18	0.0010	0.0010	0.000	0.003	0.000	0.002	0.001
Femoral muscle		0.0011	0.0010	0.000	0.004	0.000	0.002	0.001
Heart		0.0020	0.0015	0.000	0.006	0.001	0.003	0.001
Liver		0.0047	0.0025	0.000	0.030	0.001	0.004	0.007
Kidney		0.0068	0.0025	0.000	0.035	0.002	0.006	0.009

Q_1_—lower quartile; Q_3_—upper quartile; SD—standard deviation.

**Table 2:** Mercury concentrations in organs of Eurasian magpies in milligrams per kilogram of wet weight.

Organ Examined	N	Arithmetic Mean	Median	Min	Max	Q_1_	Q_3_	SD
Pectoral muscle	22	0.0029	0.0025	0.000	0.006	0.001	0.005	0.002
Femoral muscle		0.0037	0.0030	0.000	0.009	0.001	0.007	0.003
Heart		0.0064	0.0055	0.001	0.017	0.002	0.009	0.004
Liver		0.0205	0.0150	0.001	0.050	0.003	0.032	0.018
Kidney		0.0250	0.0220	0.001	0.055	0.002	0.046	0.021

Q_1_—lower quartile; Q_3_—upper quartile; SD—standard deviation.

**Table 3:** Correlation coefficients between Hg concentrations in the common woodpigeon organs analyzed.

Organ Examined	Femoral Muscle	Heart	Liver	Kidney
Pectoral muscle Femoral muscle Heart Liver	0.9789 **	0.8749 **	0.7019 **	0.8139 **
		0.8975 **	0.7153 **	0.8193 **
			0.6075 **	0.7658 **
				0.8002 **

** Correlation coefficients are significant with *p* ≤ 0.01.

**Table 4:** Correlation coefficients between Hg concentrations in the Eurasian magpie organs analyzed.

Organ Examined	Femoral Muscle	Heart	Liver	Kidney
Pectoral muscle Femoral muscle Heart Liver	0.8803 **	0.6724 **	0.6279 **	0.6362 **
		0.6603 **	0.7395 **	0.6880 **
			0.6449 **	0.6174 **
				0.9571 **

** Correlation coefficients are significant with *p* ≤ 0.01.

## Data Availability

All data generated or analyzed during the study are included in this published article. The datasets used and/or analyzed in the current study are available from the corresponding author upon reasonable request.
